# Prediction of effectiveness of potassium-competitive acid blocker and serotonin noradrenaline reuptake inhibitor on abnormal sensation in the throat: use of patient-reported outcome measures (PROMs)

**DOI:** 10.1007/s00405-020-06544-0

**Published:** 2021-01-03

**Authors:** Nao Takahashi, Kaori Ikeda, Genki Iwai, Kaori Shinbori, Hironori Baba, Takanobu Sasaki, Kuniyuki Takahashi, Yuka Morita, Arata Horii

**Affiliations:** Department of Otolaryngology Head and Neck Surgery, Niigata University Graduate School of Medical and Dental Sciences, 1-757, Asahimachi-dori, Chuo-ku, Niigata City, 951-8510 Japan

**Keywords:** Gastro-esophageal reflux disease, Pharynx, Sensation, The Glasgow Edinburgh Throat Scale

## Abstract

**Purpose:**

To determine patients with abnormal sensation in the throat (AST) who would respond to potassium-competitive acid blocker (P-CAB) or serotonin noradrenaline reuptake inhibitor (SNRI) treatment.

**Methods:**

AST patients were randomly divided into two groups. Thirty-one and 21 patients received P-CAB and SNRI treatment, respectively. GETS-J, the Japanese version of Glasgow Edinburgh Throat Scales (GETS), consisted of three subscales of throat symptoms (globus sensation, pain/swelling of the throat, and dysphagia) and somatic distress due to the disease, Frequency Scale for the Symptoms of Gastro-esophageal reflux disease (FSSG), and Hospital Anxiety and Depression Scale (HADS) were used before and after treatments. Responders to treatments were defined as those who showed 50% or more decrease in symptom scores or somatic distress.

**Results:**

Pre-treatment GETS-J pain/swelling scores and FSSG acid reflux scores were higher in P-CAB responders and decreased after treatment. Receiver operating characteristic curve for pain/swelling subscale had an area under the curve (AUC) of 0.792 to predict P-CAB responders and a score of 11 provided the best combination of sensitivity (62.5%) and specificity (80%). Somatic distress and HADS anxiety scores, but no other GETS-J symptom scores, decreased after SNRI treatment. Pre-treatment globus scores were lower in SNRI responders. AUC value for globus subscale to predict SNRI responders was 0.741 and a score of 6.5 provided the best combination of sensitivity (70%) and specificity (73%).

**Conclusions:**

Pain/swelling is a characteristic symptom in AST patients who respond to P-CAB treatment. SNRI treatment would be effective for somatic distress in cases with mild symptoms.

## Introduction

Globus sensation is defined as the feeling of a lump or something stuck in the throat [1]. In Japan, the term “abnormal sensation in the throat (AST),” i.e., an unusual or strange sensation in the throat that cannot be explained by local findings on routine inspection, is frequently used rather than “globus sensation” [2]. AST comprises a broad category of throat symptoms that includes globus sensation. Globus sensation may be a multifactorial condition that includes structural, functional, and psychogenic disorders [3–12]. Globus sensation is partly considered an extra-esophageal symptom of gastro-esophageal reflux disease (GERD), though not in all cases [13]. Therefore, proton pump inhibitors (PPIs) are used for globus patients who are suspected of having reflux disease as revealed by multichannel intraluminal pH-impedance probe testing (MII) [14]. However, MII is a relatively invasive and expensive method to perform for all globus patients. Moreover, psychologic illness such as anxiety and depression may be causally related to or exacerbate the symptoms of AST/globus sensation. In the present study, among patients with AST, we aimed to determine those who would be expected to respond to antacid treatments or anti-depressants.

For this purpose, we used three kinds of patient reported outcome measures (PROMs) before and after treatment: Japanese version of the Glasgow Edinburgh Throat Scale (GETS-J) [15], Frequency Scale for the Symptoms of GERD (FSSG) [16], and Hospital Anxiety and Depression Scale (HADS) [17].

## Materials and methods

This study was conducted under approval of the Institutional Review Board of Niigata University Medical and Dental Hospital (#2015–2505). Written informed consent was obtained from all participants.

### Patients

Fifty-two patients who complained of AST persisting longer than 1 month were enrolled in the study. Patients included 25 men and 27 women aged 58.8 ± 12.2 (mean ± SD) years. Routine inspection including endoscopic examination revealed no abnormal local findings for all patients.

### Treatment

Patients were randomly divided into two groups without patients selection criteria. An antacid, a potassium-competitive acid blocker (P-CAB), vonoprazan fumarate 20 mg/day, was administered to 31 patients for 4 weeks. A serotonin noradrenaline reuptake inhibitor (SNRI), milnacipran hydrochloride (15 mg/day) was administered to 21 patients for 4 weeks. There were no differences in sex and age between the groups (data not shown).

### Outcome measures

Three kinds of PROMs were used in this study: the GETS-J, FSSG, and HADS. The GETS-J [15] is a Japanese version of GETS [18], a 10-item questionnaire for evaluating throat symptoms comprising three subscales related to dysphagia, globus sensation, and pain/swelling in the throat, as well as somatic distress due to the disease. Each subscale consists of three questions and each question is scored on a scale from 0 (none) to 7 (unbearable). The FSSG is a 12-item questionnaire on GERD symptoms consisting of two subscales, namely acid reflux symptoms and esophageal dysmotility symptoms [16]. The HADS comprises two separate subscales for assessing anxiety and depression symptoms [17]. The GETS-J, FSSG, and HADS subscale scores were compared between pre- and post-treatment.

### Improvement ratio

The improvement ratio was defined as the ratio of the post-treatment score to the pre-treatment score (post-score/pre-score) on each subscale. The correlation of the improvement ratio with somatic distress and each subscale score was examined.

### Responders vs. non-responders

Patients were divided into two groups: responders and non-responders to treatments. Responders were defined as those who showed 50% or more decrease in scales. All other patients were defined as non-responders. 50% or more decrease was chosen based on the value used in previous studies that examined drug effects on globus sensation [19, 20].

### Predictive factors for treatment outcome

The GETS-J, FSSG, and HADS subscale scores before treatment were compared between the responders and non-responders. To evaluate the capability to predict response to treatment, receiver operating characteristic (ROC) curves were created.

### Statistics

Differences in each of the FSSG, GETS-J, and HADS subscale scores before and after treatment were tested by the paired *t* test. Correlations were tested with Pearson’s product moment correlation coefficient. Differences in each of the FSSG, GETS-J, and HADS subscale scores before treatment between the responders and non-responders were tested by Welch’s test. *p* < 0.05 was considered statistically significant in all comparisons.

## Results

### Effects of P-CAB and SNRI on GETS-J, FSSG, and HADS

**Fig. 1:**
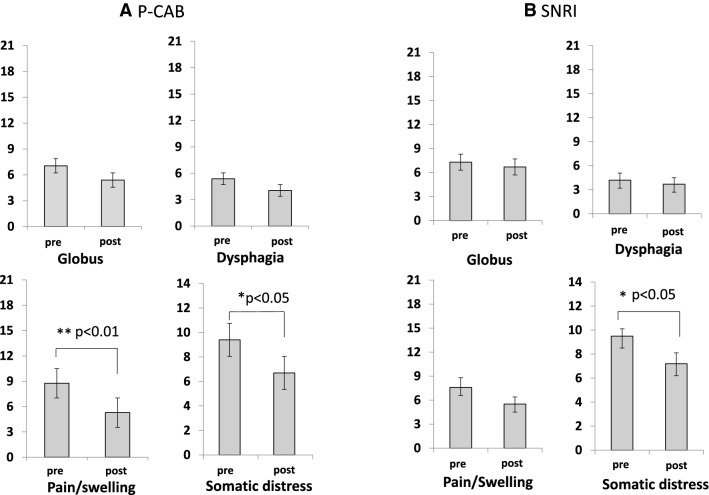
Effects of **a** P-CAB and **b** SNRI treatment on each GETS-J symptom subscale and somatic distress. **a** Scores for the pain/swelling subscale and somatic distress were significantly decreased after the P-CAB treatment. **b** Only somatic distress, and no other GETS-J symptom subscale scores decreased after the SNRI treatment

**Fig. 2:**
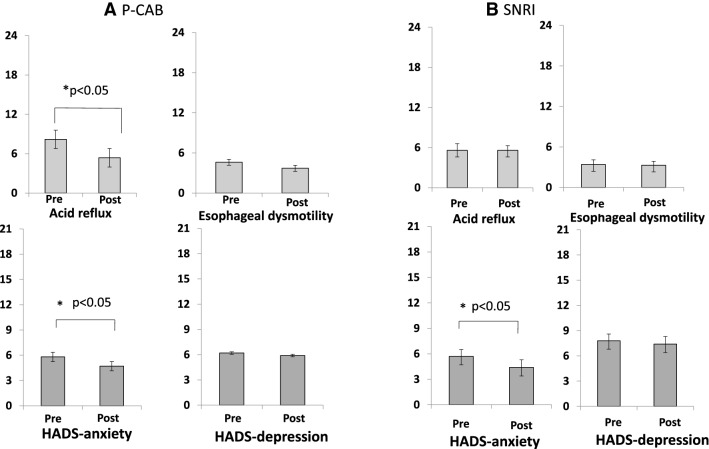
Effects of **a** P-CAB and **b** SNRI treatment on FSSG and HADS. **a** The scores of the FSSG acid reflux subscale, but not the esophageal dysmotility subscale, were significantly decreased after the P-CAB treatment. **b** There were no differences in scores of both FSSG subscales pre- and post-SNRI treatment. The scores on the HADS anxiety subscale, but not on the depression subscale, were significantly decreased after the P-CAB or SNRI treatment

The pain/swelling subscale scores among the three GETS-J symptom subscales along with the somatic distress scores were significantly decreased after the P-CAB treatment (Fig. 1a). Only the GETS-J somatic distress scores but no symptom subscale scores of GETS-J decreased after the SNRI treatment (Fig. 1b). The FSSG acid reflux subscale scores, but not the esophageal dysmotility subscale scores, were significantly lower 4 weeks after the P-CAB treatment compared to the pre-treatment scores (Fig. 2a). There were no differences in scores of both FSSG subscales between pre- and post-SNRI treatment (Fig. 2b). The scores of the HADS anxiety subscale, but not on the depression subscale, were significantly decreased after the P-CAB or SNRI treatment (Fig. 2a, b).

### Improvement ratio

**Fig. 3:**
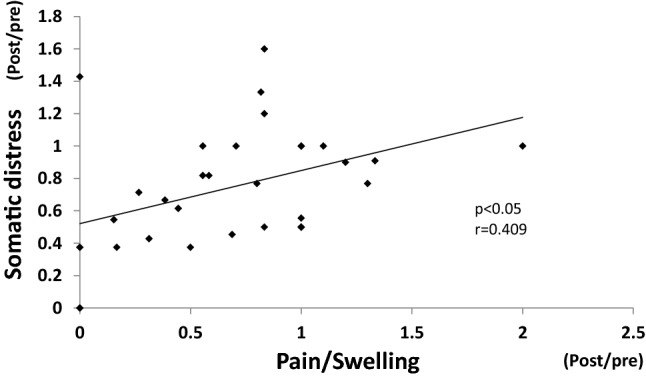
Correlation between improvement ratios (post/pre) of somatic distress and GETS-J pain/swelling subscale. Improvement ratio of somatic distress showed significant correlation with that of the GETS-J pain/swelling subscale

Among the three GETS-J symptom subscales, the scores for the pain/swelling subscale, but not the globus sensation or dysphagia subscales, decreased 4 weeks after the antacid treatment (Fig. 1a). Therefore, the correlation between the improvement ratios of the pain/swelling GETS-J subscale score and that of somatic distress was tested. Results showed that they were significantly positively correlated (*r* = 0.409, *p* < 0.05) (Fig. 3). There was no correlation between the improvement ratios of the HADS anxiety subscale score and somatic distress (data not shown).

### Responders vs. non-responders before treatment

**Fig. 4:**
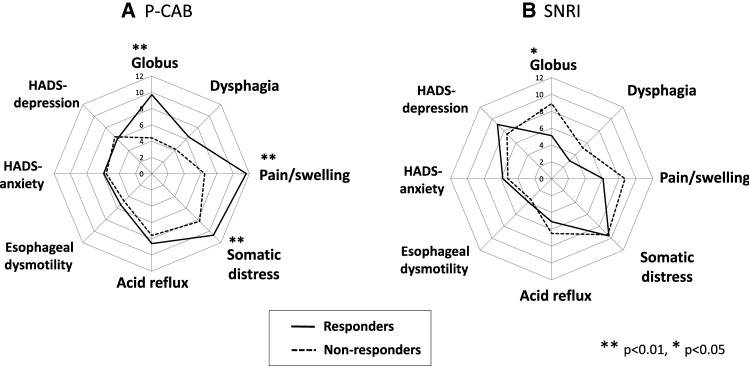
Comparison of pre-treatment status between responders and non-responders to **a** P-CAB and **b** SNRI treatment. **a** The pain/swelling, globus sensation, and somatic distress GETS-J scores were significantly higher in the responders than in the non-responders before the P-CAB treatment. **b** The scores of the GETS-J globus subscale were significantly lower in the responders than in the non-responders before SNRI treatment

Responders to the P-CAB treatment were defined as those who showed 50% or more decrease in the pain/swelling subscale score, since pain/swelling was the only symptom subscale of GETS-J that decreased after the P-CAB treatment. There were 14 responders and 17 non-responders. The pain/swelling, globus, and somatic distress GETS-J scores were significantly higher in the responders than in the non-responders before the P-CAB treatment (Fig. 4a). All other subscale scores were not significantly different between the responders and non-responders prior to the P-CAB treatment (Fig. 4a). Responders to the SNRI treatment were defined as those who showed 50% or more decrease in somatic distress. There were 10 responders and 11 non-responders. The scores of the GETS-J globus subscale were significantly lower in the responders than in the non-responders before the SNRI treatment (Fig. 4b).

### ROC curves

**Fig. 5:**
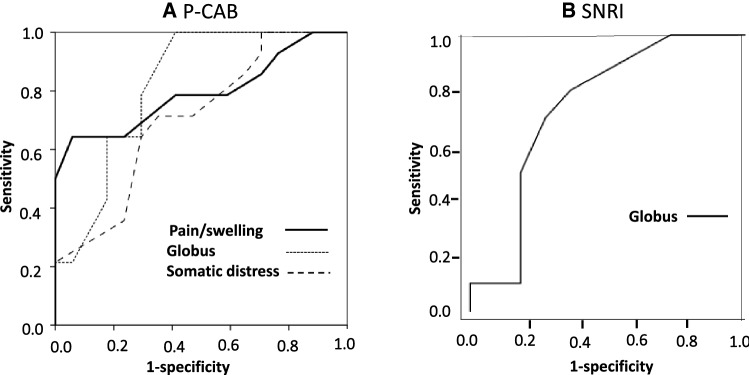
Receiver operating characteristic (ROC) curves to predict responders to **a** P-CAB and **b** SNRI treatment. **a** The area under the curve (AUC) values of the ROC curves for the pain/swelling, globus sensation, and somatic distress subscales were 0.792, 0.819, and 0.706, respectively. A score of 11 on the pain/swelling subscale provided the best combination of sensitivity (62.5%) and specificity (80%) for predicting response to P-CAB treatment. **b** The AUC of the ROC curve for the globus subscale was 0.741. A score of 6.5 on the globus sensation subscale provided the best combination of sensitivity (70%) and specificity (73%) for predicting the response to SNRI treatment

To evaluate the capability to predict response to treatment, ROC curves for the pain/swelling, globus, and somatic distress subscales, which were all higher in the responders than in the non-responders before the P-CAB treatment (Fig. 5a), were created. The results showed that the area under the curve (AUC) values for the pain/swelling, globus, and somatic distress scores were 0.792, 0.819, and 0.706, respectively (Fig. 5a). A score of 11 on the GETS-J pain/swelling subscale provided the best combination of sensitivity (62.5%) and specificity (80%) for predicting the response to P-CAB treatment. The ROC curve for the GETS-J globus subscale, which was significantly lower in the responders than in the non-responders before the SNRI treatment, had an AUC of 0.741 to predict responders to SNRI treatment (Fig. 5b). A score of 6.5 on the GETS-J globus subscale provided the best combination of sensitivity (70%) and specificity (73%) for predicting the response to SNRI treatment.

## Discussion

### Effects of antacid treatment on abnormal sensation in the throat

The 4-week P-CAB treatment significantly improved the FSSG acid reflux subscale scores (Fig. 2a), GETS-J pain/swelling subscale scores (Fig. 1a) and somatic distress due to the disease (Fig. 1a). The improvement ratio of somatic distress showed significant correlation with that of the pain/swelling subscale (Fig. 3). These results suggest that P-CAB treatment may decrease acid secretion leading to a decrease in the FSSG acid reflux score and the GETS-J pain/swelling score. Pain/swelling in the throat may be a characteristic symptom and one of the factors that cause somatic distress in patients with AST. The HADS anxiety subscale scores also decreased after antacid treatment (Fig. 2a). Given that P-CAB itself does not have any psychoactive effects, the decrease in anxiety may have resulted from alleviation of somatic distress.

There were 14 responders and 17 non-responders, indicating that the overall effective rate of the P-CAB treatment on AST was 45.1%. Pantoprazole (40 mg), one of the PPIs, for four weeks showed an effective rate of 35.7% [19] and 53.7% [20] when the same threshold to determine the responders (> 50% reduction in symptom scores) was used. Our results of P-CAB (45.1%) were comparable with these studies. As also reported previously, the effective rate of long-term PPI treatment for patients with globus sensation is approximately 44% [16]. One may argue that antacid treatment for 4 weeks is too short a duration. However, the overall effective rate of the 4 weeks P-CAB treatment in this study was comparable with that of long-term PPI treatment, perhaps because P-CABs are more potent acid suppressing agents than PPIs [21]. It is expected that the effective rate may be improved if only patients with reflux disease are selected based on MII results [14].

### Responders vs. non-responders to antacid treatment

Because the P-CAB treatment was not always effective, with an effective rate of approximately 45%, discrimination of the responders from non-responders before treatment is an important issue in terms of cost effectiveness. The GETS-J pain/swelling, globus, and somatic distress subscale scores were significantly higher in the responders than in the non-responders before treatment (Fig. 4a), suggesting that these three scales may be potential prognostic predictors of P-CAB effectiveness. ROC curves for each subscale were plotted on the same graph to compare the predictive capabilities. Among these three subscales, the AUC for pain/swelling and globus was approximately 0.8, whereas that for somatic distress was approximately 0.7 (Fig. 5a). Given that the globus subscale score did not decrease after treatment (Fig. 1a) and that the improvement ratio of pain/swelling showed significant correlation with that of somatic distress (Fig. 3), it is suggested that the pain/swelling subscale rather than the globus subscale may be more suitable as a prognostic predictor. When the cut-off point of 11 (full score 21) was adopted, the sensitivity and specificity to predict more than 50% decrease in pain/swelling symptoms (responders) with P-CAB treatment were 62.5 and 80%, respectively.

In contrast to the current results, it was reported that in patients with globus sensation and GERD, the FSSG acid reflux score before PPI treatment was higher in responders than in non-responders and that non-responders had ineffective esophageal motility on high-resolution manometry [22]. This discrepancy in the results may be due to differences in patient background (AST vs. globus sensation with GERD) and the medication used (P-CAB vs. PPIs).

### Effects of SNRI treatment on abnormal sensation in the throat

AST and globus sensation may be multifactorial conditions that include structural, functional, and psychological disorders [3–12]. Indeed, it was reported that psychosocial stress induced constriction of the esophageal wall [23] and that depression had significant effects on prognosis of GERD [24]. In the present study, SNRI treatment for 4 weeks significantly improved the HADS anxiety subscale (Fig. 2b) and somatic distress due to the disease (Fig. 1b). On the contrary, SNRI treatment had no effect on the other GETS-J symptom subscales (Fig. 1b). SNRI treatment differed from antacid treatment in that it did not show direct effects on throat symptoms; however, it decreased patients’ somatic distress perhaps by reducing anxiety.

### Responders vs. non-responders to SNRI treatment

There were 10 responders and 11 non-responders, indicating that the overall effective rate of SNRI on AST was 48%. The GETS-J globus subscale was significantly lower in the responders than in the non-responders before SNRI treatment (Fig. 4b), suggesting that the globus subscale may be a potential prognostic predictor of SNRI effectiveness. The ROC curve for the globus subscale has an AUC of 0.741 (Fig. 5b) showing moderate accuracy. When the cut-off point of 6.5 (full score 21) was adopted, the sensitivity and specificity to predict responders to SNRI treatment were 70% and 73%, respectively. SNRI affected the somatic distress in mild cases.

### Selection of antacid agents or SNRIs in treating AST

Laryngopharyngeal reflux may be one of the major causes of globus sensation and AST. Therefore, antacid agents are often used for these conditions. In treating suspect laryngopharyngeal reflux disease (LPRD), up-front, pH-impedance, and manometry testing are superior to empiric PPI trials in terms of minimizing cost, although the latter are still common [25]. Although ideal, it would be unrealistic to perform pH-impedance and manometry testing for all AST patients. Alternatively, based on the present results, we suggest that the GETS-J pain/swelling subscale can predict responders to antacid treatment among patients with AST. A score of 11 showed high specificity (80%) in predicting the response to treatment, whereas the sensitivity was relatively low (62.5%). Therefore, if a patient with AST has a score lower than 11 on the pain/swelling subscale, P-CAB treatment is not recommended.

Although SNRI treatment did not show direct effects on throat symptoms, it was beneficial in relieving patients’ somatic distress perhaps through decrease in anxiety. However, its effects on somatic distress were limited to patients with mild globus sensation: score of 6.5 had sensitivity (70%) and specificity (73%) to predict responders. Therefore, SNRI treatment may be recommended for AST patients with milder throat symptoms with globus sensation subscale scores of 6.5 or less. Nonetheless, we cannot exclude the possibility that these effects on somatic distress might be non-specific ones, since there was no correlation between the improvement ratios of somatic distress and HADS anxiety subscale.

## Conclusions

Pain/swelling is a characteristic symptom in patients with AST who respond to P-CAB treatment. A score of 11 showed high specificity (80%) in predicting the response to treatment, whereas the sensitivity was relatively low (62.5%). SNRI treatment would be effective for somatic distress in cases with mild symptoms whose globus sensation subscale scores are 6.5 or less.
